# Designing Chatbots to Treat Depression in Youth: Qualitative Study

**DOI:** 10.2196/66632

**Published:** 2025-06-19

**Authors:** Florian Onur Kuhlmeier, Luise Bauch, Ulrich Gnewuch, Stefan Lüttke

**Affiliations:** 1 Human-Centered Systems Lab, Institute for Information Systems (WIN), Karlsruhe Institute of Technology, Karlsruhe, Germany; 2 Department of Clinical Psychology and Psychotherapy, Institute of Psychology, University of Greifswald, Franz-Mehring-Straße 47, Greifswald, 17489, Germany, 49 38344203723; 3 Chair of Explainable AI-Based Business Information Systems, School of Business, Economics and Information Systems, University of Passau, Passau, Germany; 4 Department of Clinical Psychology and Psychotherapy, University of Tübingen, Tübingen, Germany

**Keywords:** human-centered design, depression, mental disorder, mental health, emotional health, youth, teenager, depressive, digital mental health intervention, questionnaire, semi-structured interview, think-aloud, chatbot prototype, remitted depressive, mixed-method, mobile phone

## Abstract

**Background:**

Depression is a severe and prevalent mental disorder among youth that requires professional care; however, various barriers hinder access to effective treatments. Chatbots, one of the latest innovations in the research on digital mental health interventions, have shown potential in addressing these barriers. However, most studies on how to design chatbots to treat depression have focused on adult populations or prevention in the general population.

**Objective:**

This study aimed to investigate the problems faced by youth with depression and their adaptive coping strategies, as well as attitudes, expectations, and design preferences for chatbots designed to treat depression.

**Methods:**

We conducted a qualitative study, consisting of a semistructured interview and a concurrent think-aloud session, in which participants interacted with a chatbot prototype with 14 youth with a current or remitted depressive episode.

**Results:**

The participants reported a wide range of problems beyond core depressive symptoms, such as interpersonal challenges, concerns about school and the future, and problems with human therapists. Adaptive coping strategies varied, with most seeking social support or engaging in pleasant activities. Attitudes toward chatbots for depression treatment were predominantly positive, with participants expressing less anxiety about using a chatbot than about seeing a human therapist. Participants showed diverse and partially contradictory design preferences, which included diverse dialogue topics, such as discussing daily life, acute problems, and therapeutic exercises, as well as various preferences for personality, language use, and personalization of the chatbot.

**Conclusions:**

Our study provides a comprehensive foundation for designing chatbots that meet the unique needs and design preferences of youth with depression. These findings can inform the design of engaging and effective chatbots tailored to this vulnerable population.

## Introduction

Depression is a prevalent mental disorder in youth with significant personal and socioeconomic consequences that requires professional care [1,2]. Despite the availability of effective treatments, such as cognitive behavioral therapy (CBT) [3], accessing them remains challenging. Even when professional services are free and accessible [4], many youth avoid seeking professional support due to perceived stigma, a preference to solve problems by themselves, and fear of psychotherapeutic settings [5-8]. Interestingly, such attitudinal barriers may be more important reasons for not seeking help than structural barriers, such as limited treatment resources and long waiting periods [8]. Digital mental health interventions (DMHIs) are a promising and effective way to overcome these barriers because they provide anonymous and self-empowered access to effective professional care [9]. However, to leverage the full potential of DMHIs in the treatment of depressive symptoms in youth, 2 major limitations need to be overcome,that is, low adherence [10] and difficulty in establishing a therapeutic alliance, which is viewed as a crucial factor for effective psychotherapy [11].

Chatbots, software systems that interact with users using natural language [12], have shown the potential to address these limitations. They are well accepted, feasible, and have shown promising effectiveness in strengthening mental health [13-16]. In addition, incorporating chatbots into DMHIs improves user engagement [17] and mental health outcomes [18]. Notably, users seem to develop a therapeutic alliance with chatbots [19-21] partly because of social cues such as empathetic messages and humor [15,22]. Despite these encouraging results, most studies on mental health chatbots, including those targeting depression symptoms, have focused on adult populations [13-16,23]. This is a key shortcoming because the results from adult populations cannot be generalized to youth. Youth face significant developmental changes in their biological, psychological, and social systems [24], and depression symptoms differ from those in adulthood, especially at the onset of puberty [25]. Furthermore, youth interact with smartphones and chatbots differently than adults [26,27], and have expressed that existing DMHIs often fail to address their specific concerns adequately [28].

To address this research gap, recent studies have explored the design of chatbots for youth mental health. Høiland et al [29] involved youth in designing a chatbot for high-school health services aimed at preventing mental disorders. Through focus groups, they identified four key support needs: (1) receiving information about mental health, (2) relating to someone beyond their immediate network, (3) receiving support for self-help, and (4) being referred to mental health services. Similarly, Grové [30] developed a preventive mental health chatbot with high-school students. Participants suggested topics such as school, family, friends, sexuality, and identity as well as resources for adaptive coping strategies, mindfulness, and distractions. They expressed a preference for chatbots with inspiring, charismatic, and fun personalities using emojis, humor, and GIFs. While these 2 studies provide valuable insights, they focused on the prevention rather than the treatment of mental disorders and addressed a broad spectrum of mental health issues rather than specifically targeting depression. Thus, there is a critical need for research on how to design chatbots that focus on specific problems of youth with depressive symptoms to achieve sufficient engagement and optimal treatment outcomes.

Our study aims to address this research gap by investigating the following research questions: (1) What problems do youth with depressive symptoms face? (2) What adaptive coping strategies do they apply? (3) What attitudes and expectations do they have for chatbots designed to treat depression among youth? (4) What are their design preferences?

By addressing these questions, we aim to provide a comprehensive foundation for developing chatbot-based DMHIs tailored to the unique needs and preferences of youth with depression. This foundation will facilitate the development of engaging and effective DMHIs for this vulnerable population.

## Methods

### Study Design

We conducted a qualitative study to examine how to design a chatbot to treat depression in youth. The study included a questionnaire, a semistructured interview, and a concurrent think-aloud session with a chatbot prototype. We chose interviews because they allowed us to gather rich, detailed data on participants’ problems, coping strategies, attitudes and expectations, and chatbot design preferences.

### Participants

Participants were eligible if they were between 14 and 17 years of age, owned a smartphone, and met diagnostic criteria for a current or remitted depressive episode. Participants with suicidal ideation or psychotic symptoms were excluded. Participants were recruited through a resident child and adolescent psychiatrist and the University’s newsletter between June and August 2021.

The target sample size was calculated based on the goal of data saturation [31], considering age group (14‐15 and 16‐17 y), depression status (remitted and acutely depressed), and gender (women and men), assuming homogeneity within each subgroup. Given that data saturation can be achieved at 6 cases per homogeneous group [31], the study aimed to recruit 48 participants. The sample was recruited through convenience sampling but the sampling was constrained by the predetermined recruitment period and available recruitment channels. The final sample consisted of 14 participants (12 women and 2 nonbinary).

### Procedure

After participants and caregivers provided informed consent, we assessed their eligibility to participate in the study. Next, enrolled participants answered a questionnaire on sociodemographic information and mental health on a laptop. We conducted semistructured interviews to explore participants’ experiences with depression, their adaptive coping strategies, attitudes and expectations toward chatbots for depression, and their design preferences. Finally, the participants interacted with a prototype chatbot using the concurrent think-aloud method.

### Material

#### Eligibility Interview

We conducted a semistructured interview to evaluate participants’ eligibility to participate in the study. The interview guide included questions regarding age, smartphone ownership, suicidal ideation, and symptoms of depression, and psychotic disorders. The questions on suicidal ideation and symptoms of depression and psychotic disorders were based on the *Diagnostic and Statistical Manual of Mental Disorders, Fifth Edition* (*DSM-5*) criteria [32] and Kiddie-Schedule for Affective Disorders and Schizophrenia Present and Lifetime [33]. The complete eligibility interview is presented in Multimedia Appendix 1 .

#### Questionnaire

Participants completed a questionnaire on demographic characteristics (age, gender, and level of education), history of mental disorders, and previous experience with psychotherapy. We assessed the prevalence of current symptoms of depression using the 8-item Patient Health Questionnaire (PHQ-8) [34] as well as current symptoms of anxiety using the Screen for Child Anxiety Related Disorders (SCARED) [35]. To complement the insights from the semistructured interview on the design preferences, participants answered a questionnaire on potential chatbot capabilities. This questionnaire is based on CBT manuals and literature on the content of DMHIs for depression [36-38] and comprises 13 capabilities that a chatbot to treat depression in youth could implement. For each capability, we asked participants to indicate the extent to which the chatbot should support them on a scale from 1 (“strongly disagree”) to 5 (“strongly agree”). The capabilities are presented in Textbox 1. The participants completed all questionnaires on a laptop using SoSci Survey (SoSci Survey GmbH).

Textbox 1. **Items listed in the questionnaire on chatbot capabilities.**

The chatbot should support me with…

- how I can become more physically active or do sports.
- how to sleep better.
- how I can change negative or self-critical thinking.
- how I can do more activities that are important to me or that I have enjoyed in the past.
- tracking my mood.
- learning more about my depression.
- improving my social skills.
- reminding me to take my medication.
- connecting with an expert (eg, psychotherapist) if I feel very bad.
- connecting with other people who have similar problems.
- how I can use my breath to make me feel better.
- writing a journal with things that concern me or for which I am grateful for.
- writing about events from my life.

#### Study Interview

We conducted semistructured interviews to investigate the problems participants faced due to depression, their adaptive coping strategies, their attitudes and expectations toward chatbots for depression, and their chatbot design preferences. Table 1 provides an excerpt of the interview guide. We asked several questions about each topic and prepared subquestions to follow up on specific details or offer suggestions based on the participants’ initial responses.

**Table 1.** Excerpt from the interview guide (translated from German).

Topic	Question
Problems	Think of a period where you felt down. How did it look like?
Adaptive coping strategies	What have you tried in the past to feel better?
Attitudes and expectations towards therapy chatbots	Do you know what a chatbot is? What do you think it would be like for you to use a chatbot that is there to help you with one of the problems you have mentioned? For example, what could be the advantages and disadvantages?
Design preferences for therapy chatbots	Imagine you are thinking about using one of these chatbots. How should it need to be designed, so that you would download and use it? How do you imagine an ideal conversation with the chatbot? For example, what topics would you like to discuss or what its personality should be like? If it was accessible as a mobile app, what should the app look like? (What) Would you like to personalize? How often would you like to use such a chatbot (per week)? How long should every session last?

#### Chatbot Prototype: Cady

**Figure 1.**
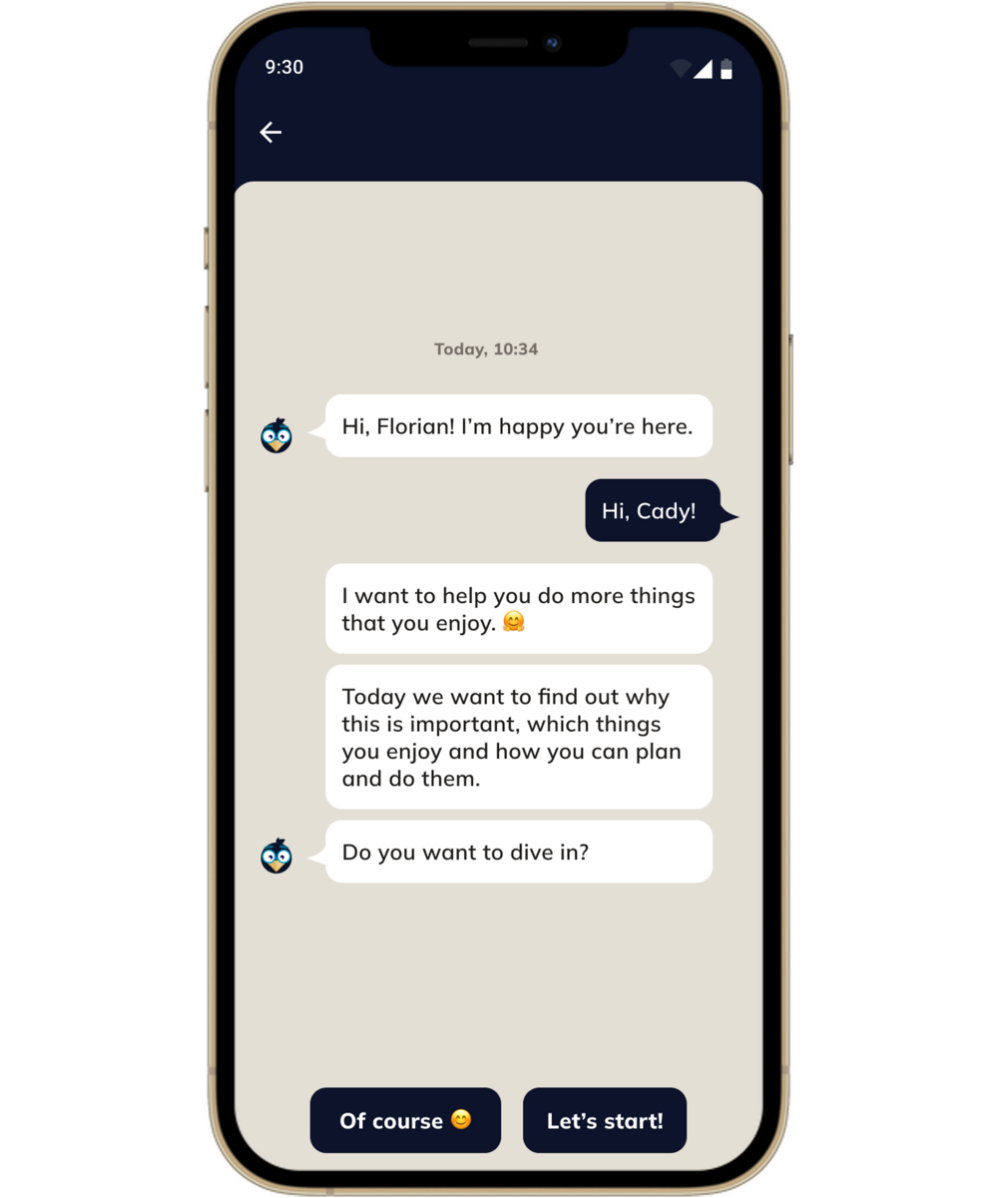
Screenshot of an example conversation between Cady and a participant.

Cady is the prototype of a chatbot for the treatment of depressive symptoms in youth that we developed for this and subsequent studies. Cady guides participants through a behavioral activation exercise, which is a core component of CBT to treat depression in youth [3]. Behavioral activation aims to encourage patients to engage in pleasant activities to overcome positive reinforcement deficits [39]. The conversation comprised the following sections: (1) introduction; (2) mood check on a scale from 1 (lowest) to 5 (highest) with adaptive empathetic responses; (3) psychoeducation on the relationship between behavior, thoughts, and feelings; (4) finding pleasant activities; (5) planning pleasant activities; (6) advice on how to overcome barriers when performing activities; and (7) feedback and goodbye. The conversation was designed by licensed psychologists based on established CBT manuals for youth [40,41] with a focus on positive, activating, and age-adequate language style. Cady also used emojis in its messages and sent GIFs. Figure 1 represents a screenshot of an example conversation between Cady and a participant. We named the chatbot Cady in accordance with the title of our research project and did not specify its age, gender, or other demographic characteristics to prevent specific demographic characteristics from influencing the results. We developed Cady using the prototyping software, Botsociety [42]. The chatbot was built with a rule-based architecture, where each user input triggers a predetermined response pathway following a decision tree structure. To ensure appropriate responses and maintain therapeutic quality, users primarily interacted with the chatbot by selecting from predefined options presented as buttons. We allowed free-text input in specific situations, for example, to identify and schedule pleasant activities. However, these free-text responses did not alter the chatbot’s conversation pathway.

#### Think-Aloud

We applied the concurrent think-aloud method [43], in which participants are asked to express their thoughts and feelings loudly while interacting with an app. At the beginning of the think-aloud session, the interviewer introduced the chatbot prototype Cady to the participants and explained the think-aloud method. Subsequently, participants interacted with Cady using a laptop. While the participants interacted with the prototype, we recorded the laptop screen and their comments. The interviewer stayed in the room because all participants felt it was easier to share their thoughts and feelings when the interviewer was in the room. The interviewer asked for further explanation if the statements were unclear or nonspecific.

### Data Analysis

The interview and think-aloud session were recorded using an audio recorder (Zoom Q2n [Zoom Corporation]) and were transcribed verbatim. Furthermore, 2 coders analyzed the semistructured interviews and concurrent think-aloud sessions using qualitative content analyses by Mayring and Fenzl [44] and Mayring [45] with the QCAmap software (Fenzl and Mayring) [46]. Similar to Zehetmair et al [47], we chose an inductive coding approach to achieve the most unbiased and thorough description of the data, which we deemed important for the exploratory nature of our study [44,45]. Both coders (Lilli Feline Metelmann and LB) coded the material independently, discussed their coding results, and jointly developed a category system. When disagreements between the coders could not be resolved, a third coder (FOK) was consulted. We chose this process because intercoder comparison is not feasible with inductive category development [44,45]. In an inductive approach, categories are created bottom-up from the material rather than selected before data analysis, as in deductive approaches [44,45]. We report the frequencies of the categories and codes to increase transparency and demonstrate the saliency of the codes in the data. The category system is provided in Multimedia Appendix 2. The questionnaires were analyzed using R (version 4.1.3; R Core Team). For PHQ-8, SCARED, and the potential capabilities questionnaire, we calculated descriptive statistics (mean and SD). For the potential capabilities, we also generated frequency distributions to visualize the response patterns.

### Ethical Considerations

The Institutional Review Board of the Medical Faculty of the University of Tübingen approved the study (project ID 595/2021B01). The participants and caregivers provided written informed consent (Multimedia Appendix 3) before screening for eligibility and enrollment. All participant data were deidentified before analysis by removing personal identifiers such as names, addresses, and contact information, and replacing them with unique study codes to ensure participant confidentiality. After the study, participants received a reimbursement of 30€ (equivalent to US $33) for taking part in the study. The study lasted between 1 and 2 hours.

## Results

### Participant Characteristics

In total, 14 youth between 14 and 17 years old (mean 16.1, SD 1.14 years) participated in the study. Furthermore, 12 participants identified as women and 2 as nonbinary. All participants reported a current or remitted depressive disorder. In addition, 10 participants had a PHQ-8 score of 10 or higher, indicating acute depressive symptoms [48]. Of the total, 12 participants had a SCARED score of 25 or higher, indicating current symptoms of anxiety [49]. Furthermore, 9 participants showed symptoms of both depression and anxiety. Out of 14, 9 (64%) participants were currently receiving psychotherapy, whereas 8 (57%) had received psychotherapy in the past. A comprehensive overview of the participant characteristics is illustrated in Table 2.

**Table 2.** Participant characteristics.

Characteristic	Statistical values
Age (y), mean (SD)	16.1 (1.14)
Gender, n (%)	
Women	12 (86)
Nonbinary	2 (14)
PHQ-8^a^ (sum score)	
Mean (SD)	13.57 (5.58)
≥Cut-off (10), n (%)	10 (71)
SCARED^b^ (sum score)	
Mean (SD)	40.86 (17.55)
≥Cut-off (25), n (%)	12 (86)
Psychotherapy experience, n (%)	
No experience	2 (14)
Current (≤6 mo)	9 (64)
Remitted (>6 mo)	8 (57)
Both current and past	6 (43)
Diagnosis of depression, n (%)	
Current (≤6 mo)	8 (57)
Remitted (>6 mo)	10 (71)
Recurrent (minimum 2 episodes)	5 (36)

a PHQ-8: 8-item Patient Health Questionnaire.

b SCARED: Screen for Child Anxiety Related Disorders.

### Research Question 1: Problems With Depression

All participants (N=14, 100%) experienced problems categorized as depressive symptoms, including lack of motivation and energy, depressed mood, and self-doubt. Furthermore, 2 participants explained their main problems:

*I just don’t have the strength to do anything*” and “*I’m just tired of everyday life, or of all these everyday activities like brushing my teeth.*

Some participants (9/14, 64%) reported comorbidities, such as panic attacks, anxiety, and excessive alcohol consumption, while 4 participants (28%) reported physical problems, such as tension, headaches, or stomach pain. All 14 participants (100%) also reported interpersonal problems, particularly withdrawal from social relationships, stress caused by parents, and problems with friends. Furthermore, 11 participants (78%) expressed concerns about school or their future. They shared negative experiences at school and experienced pressure to plan their future, which were reflected by statements like:

*When it comes to school, I have a very, very big fear of the future*.

*Success is a big topic right now, because of the end of school year grades.*

In total, 6 participants (42%) reported experiencing stigma associated with depression. They mentioned that adults stigmatized or trivialized their problems or feared negative reactions when sharing their problems. One participant was concerned that disclosing that they are seeing a psychotherapist would lead to others perceiving them as “sick in the head.” Most participants reported encountering difficulties with mental health care. Furthermore, 12 individuals (85%) reported experiencing treatment barriers, including attitudinal and structural barriers. Attitudinal barriers included fear of being judged, self-assessment that problems are not severe enough to deserve support, and negative experiences during psychotherapy (n=9, 64%). Participants reported ineffective techniques, difficulty with disclosing personal information, pressure to perform, trust violations, and condescending treatment from therapists. Structural barriers reported are long waiting lists or being deemed not sufficiently severe by healthcare providers or parents to qualify for professional support.

### Research Question 2: Adaptive Coping Strategies

All 14 participants (100%) identified social support as an adaptive coping strategy. The majority received social support from friends, family members, partners, teachers, or people they interacted with online. In total, 13 participants (93%) engaged in activities to distract themselves or to have a positive experience such as spending time outdoors or consuming media. A total of 9 participants (64%) reported receiving professional treatment and 9 (64%) used cognitive strategies, including positive self-talk, self-reflection, and reducing rumination. Furthermore, 6 participants (42%) implemented a daily structure including forming habits, getting up early, scheduling positive activities, and setting goals. In addition, 7 (50%) prioritized their needs as a coping mechanism, such as intentionally allocating time to self-care or avoiding stressful social situations. Of the total, 3 individuals (21%) engaged in mindfulness practice, such as breathing techniques and meditation, and 2 individuals (14%) sought online information regarding their depressive symptoms, interrelated problems and further information.

### Research Question 3: Attitudes and Expectations Toward Chatbots to Treat Depression

Most participants had positive attitudes toward and expectations of chatbots to treat depression. In total, 12 participants (85%) stated that they would be less anxious about using a chatbot than seeing a human therapist. They pointed out that using a chatbot would be a suitable option for discussing sensitive topics that they would not share with others, primarily because they would not fear negative reactions. In addition, texting was considered less intimidating than speaking with a therapist. Furthermore, 11 participants (79%) pointed out the unlimited capacity and flexibility of chatbots:

*I think it’s also an advantage that you can really chat with it at any time, because in therapy you just have one appointment per week. If you feel bad in the evening or at night or something, then you can still text the chatbot*.

In addition, participants indicated that a chatbot is more accessible and requires less effort than seeing a human therapist. A total of 8 participants (57%) expressed confidence in the effectiveness of chatbots for the treatment of depression. They indicated that such a chatbot would be capable of addressing a wide range of issues, and welcomed the idea of having a helpful everyday chatbot. All participants demonstrated keen interest in using a chatbot to treat depression either because they expected it to be effective or because they were curious about using it. One participant stated that a daily usable chatbot could alleviate feelings of loneliness. Several others noted that a chatbot’s personal and human-like nature would increase the motivation to use it, particularly compared with other less interactive DMHIs.

However, participants also reported several concerns regarding the use of chatbots to treat depression. Some participants (n=9, 64%) were skeptical about the chatbot’s intelligence and natural language capabilities. They expressed concern that the chatbot would not be able to address individual, diverse, or unusual problems effectively, and feared being disappointed if the chatbot was unable to do so. Participants were particularly worried about inappropriate answers to emotional and intimate topics or inappropriate advice for their problems. Some participants (n=9, 64%) were concerned that a conversation with the chatbot would not feel like a conversation with a human therapist, due to a potential lack of emotional intelligence or overly robotic or analytical responses. However, chatbots that appeared too human were believed to be uncanny. In total, 2 participants (14%) were skeptical about sufficient data security and privacy. Finally, 2 participants (14%) worried that their symptoms of depression, such as little motivation or forgetfulness, would result in low engagement with the chatbot, thereby preventing them from effectively using it. They also pointed out that the lack of social pressure when using a chatbot, compared to seeing a human therapist, could contribute to low engagement, which might not be solvable by the chatbot.

### Research Question 4: Chatbot Design Preferences

#### Category 1: Dialogue Topics and Therapeutic Content

All participants (n=14, 100%) shared preferences regarding dialogue topics that the chatbot should be able to cover. These suggestions include comprehensive assessments of depressive symptoms and interrelated problems, psychoeducation, therapeutic exercises that address specific issues, reminders to address basic needs, support for regulating emotions, tackling intrusive thoughts, and being distracted when needed. Furthermore, they emphasized the importance of discussing their daily lives and, more specifically, sharing current problems and receiving suggestions on how to resolve them. One participant highlighted the importance of the chatbot explicitly asking the user what type of support they require, such as emotional support (ie, listening and validation) or solution-oriented support:

*Getting advice on how to solve a problem isn’t always helpful, even if it’s well-intentioned*. […] *It would be important for the chatbot to ask or understand if you want advice or if you only want to share your feelings*.

Others elaborated on how they imagined talking about daily life and receiving advice:

*I would just talk about the things that depress me at the time, to which I don’t know the answer*.

*I would probably just chat about everyday situations that were unpleasant to me or something like that*.

**Figure 2.**
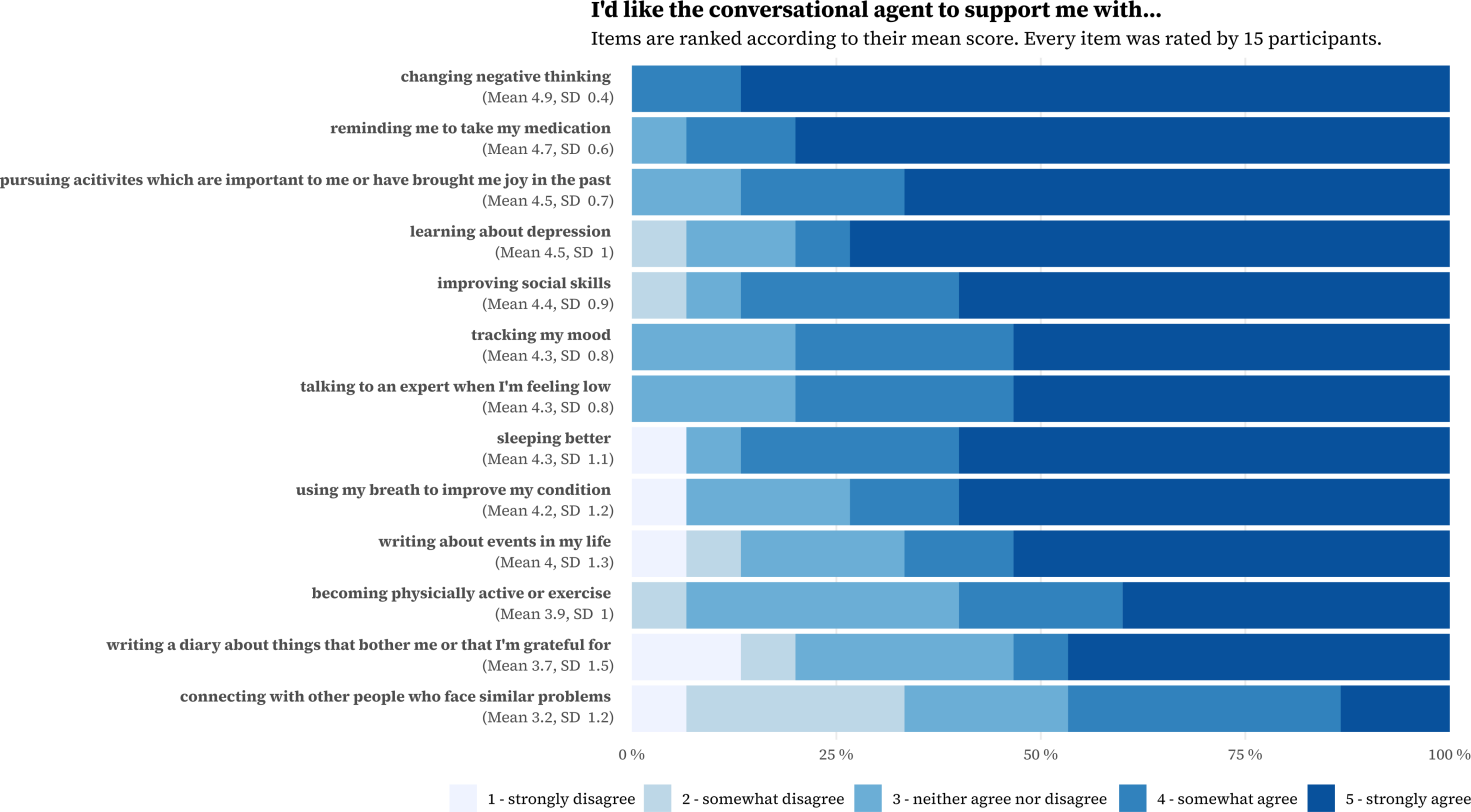
Results for how much support participants would like for several proposed chatbot capabilities.

These preferences were complemented by responses to a questionnaire regarding the chatbot’s desired capabilities. Figure 2 presents the full results. The responses indicate the significance of three key components of CBT: (1) cognitive restructuring (“changing negative thinking;” mean 4.9, SD 0.4), (2) behavioral activation (“pursuing activities that are important to me or have brought me joy in the past;” mean 4.5, SD 0.7), and (3) psychoeducation (“learning about depression;” mean 4.5, SD 1). In addition, participants expressed a preference for “improving social skills” (mean 4.4, SD 0.9). On the other hand, therapeutic writing, represented by “writing about events in my life” (mean 4, SD 1.3) and “writing a diary about things that bother me or that I’m grateful for” (mean 3.7, SD 1.5), was rated lower than most items. Finally, “connecting with other people who face similar problems” was ranked the lowest (mean 3.2, SD 1.2).

#### Category 2: Personality, Interaction Style, and Social Role

Participants made various suggestions for the chatbot’s personality (n=14, 100%), such as understanding, caring, friendly, emphatic, encouraging, humorous, interested, neutral, and nonjudgmental. During the think-aloud sessions, most participants characterized Cady’s personality as one of its main strengths. One participant said:

*I like that you communicate in a friendly way, like an internet friend.*

Furthermore, 2 participants were pleased that Cady was interested and asked them personal questions. For example, 1 participant approved that Cady had asked if she had experienced becoming less active herself:

*I just think it’s good that she is asking if I know something like that or haven’t experienced it yet.*

In terms of the interaction style and language used by the chatbot, participants suggested that it should be appropriate for the user’s age without complicated terms. This issue was also raised during think-aloud sessions. One participant asked for more age-specific language, such as short sentences:

*So, this was a therapeutic chat, but if it is supposed to be like a friend, I think it is better if it is writes like people my age, eg using short sentences*.

Another participant perceived the language as too therapeutic:

*It was relatively authentic, but you also notice that it is not written for my age*.

The human-likeness of the chatbot was also a significant factor, as participants found it important to react to feelings and to understand irony and humor. However, participants also expressed concerns about the chatbot being too human-like, which could lead to irritation or fear. During the think-aloud sessions, 1 participant appreciated that Cady disclosed personal information such as enjoying chats with nice people, which resembled a human-like trait.

Regarding the social role, the participants (n=14, 100%) held divergent views: some desired the chatbot to resemble a friend, whereas others preferred it to assume the role of a therapist or a combination of both. One participant explained their preference for a therapist-like role:

*I actually think it’s better if it were like a therapist [...], because with friends you don’t necessarily want talk about everything*.

One participant proposed using situation-specific roles:

*A therapist if you’re doing therapeutic exercises and a friend if you just want to talk*.

#### Category 3: Personalization

Personalization frequently discussed the importance of personalization (n=13, 92%). Participants reported various aspects that should be personalized, including the chatbot’s personality, gender, avatar, message style, dialogue topics, therapeutic content, the mobile app’s color theme, notifications, and user profile picture and username. However, the participants had divergent opinions on who should control the personalization of the chatbot. While some suggested that the user should be in control, others believed that the chatbot itself should control personalization. Others, in turn, proposed a mixed approach in which both the user and the chatbot share control. Some participants preferred personalization to occur only once at the beginning, whereas others preferred dynamic personalization, where personalization occurs continuously. In addition to these explicit preferences, personalization also emerged from diverging design preferences. All participants expressed a preference for an appealing user interface, but they had differing opinions on the specific design elements, with some suggesting a bright and colorful design, whereas others preferred a plain and minimalistic design with black and white colors. Most participants preferred the chatbot as a standalone app, but some preferred integration into a popular instant messaging app such as WhatsApp (Meta). Regarding the interaction modalities, most participants preferred a chatbot over a voice agent. In terms of text input, the participants believed that a mix of predefined answers and free-text input was optimal, as it combines the minimal effort of clicking predefined answers with the flexibility of unrestricted text input. In total, 2 participants (14%) expressed concerns about personalization, stating that personalized content could lead to avoidance behavior, in which users avoid topics that they find difficult or uncomfortable. They also believed that personalization of the chatbot’s appearance, such as its gender or avatar, could reduce its seriousness as a tool for treating depression.

Table 3 summarizes key chatbot design recommendations derived from results across all 4 research questions.

**Table 3.** Key design recommendations.

Topic	Design recommendation
Assessment	Incorporate repeated multidimensional assessment, covering depressive symptoms and related problems, to monitor and understand the key issues a user faces. Ensure assessments are comprehensive yet concise to minimize the effort for users.
Scope and Limitations	Communicate the chatbot’s scope and limitations clearly, for instance, when it cannot properly handle a topic. Redirect users to external resources (eg, helplines and professional interventions) in such cases to ensure users receive appropriate support.
Dialogue content	Curate a diverse content database covering a wide range of topics that are relevant to youth with depression. Integrate evidence-based therapeutic techniques alongside user-preferred coping strategies (eg, behavioral activation, cognitive restructuring, and social support). Enable the chatbot to engage in conversations about users’ daily life, including discussions on urgent problems. Support follow-up discussions on user-reported problems, track progress, and encourage reflection on how successful they carried out the learned techniques.
Personality, social role and language style	Design a chatbot with an understanding, caring, friendly, empathic, encouraging, humorous, interested, and nonjudgmental personality. Ensure the chatbot uses age-appropriate language without complicated terminology. Balance human-likeness (eg, recognizing feelings, irony, humor) with clear boundaries to avoid unrealistic or overly human-like interactions.
Personalization	Personalize the content selection and presentation. Personalize the chatbot’s persona (eg, personality, social role, gender, avatar, message style). Explore the impact of persona personalization (eg, switching between formal and casual depending on user feedback, interaction style preferences and situation). Explore the impact of who controls personalization (the user, the chatbot or both) and when or how often personalization is performed (initial vs ongoing). Ensure personalization enhances user experience while maintaining the chatbot’s therapeutic credibility and seriousness.

## Discussion

### Primary Findings

This study explored the problems youth face with depression, their adaptive coping strategies, and their attitudes and design preferences for chatbots to treat depression. Our findings indicate that youth experience diverse problems beyond core depressive symptoms, employ a range of coping strategies, hold predominantly positive attitudes toward chatbots for depression, and express diverse, sometimes contradictory, design preferences. In the following section, we discuss the results according to the four research questions guiding this study: (1) the problems youth with depression face, (2) their adaptive coping strategies, (3) their attitudes and expectations toward chatbots to treat depression, and (4) their design preferences for chatbots to treat depression. As presented in Table 3, our findings across all research questions offer specific guidance for designing chatbots tailored specifically to youth experiencing depression.

### Problems of Youth With Depression

First, participants reported various problems with depression beyond the symptoms described in the DSM-5 [50], including mental health and somatic comorbidities, interpersonal issues, and concerns about school and the future. These findings align with previous research showing that youth with depression face an increased risk for anxiety disorders, substance abuse, and physical health issues [51], as well as social withdrawal, lack of friendship [52], diminished academic achievement, school dislike, and pessimism [52]. To address these problems, engaging and effective chatbots should (1) assess both core depressive symptoms and related problems repeatedly to inform therapeutic decisions and strengthen the therapeutic relationship by understanding the users’ individual challenges, and (2) curate a diverse content database to address the wide range of problems, aligning with Li et al [53]. Second, participants reported significant barriers to receiving professional treatment, including poor mental health literacy (eg, awareness of symptom severity), attitudinal (eg, perceived stigma), and structural barriers (eg, waiting times). These findings align with research showing that youth struggle to recognize symptoms [54], hesitate to disclose personal information to professionals due to fear of judgement or not being taken seriously [7], and question the effectiveness of professional treatment [7], a concern supported by evidence that 60% of youth with depression do not benefit from psychological treatments [55]. To address these barriers, chatbots should (1) encourage emotional disclosure by providing a nonjudgmental conversational environment, something DMHIs cannot fully achieve due to their static, nonconversational design, and (2) clearly communicate their scope and limitations to set realistic user expectations and direct to external support when needed.

### Adaptive Coping Strategies

Participants reported various adaptive coping strategies, including seeking social support, establishing positive activities, using cognitive strategies, and receiving professional treatment. Many also emphasized the importance of focusing on their needs and establishing a daily structure. Most of these strategies fall into the CBT strategies behavioral activation, cognitive restructuring, and problem solving [56], which are effective and first line treatments for depression in youth [57].

The prevalence of CBT-based coping strategies may be due to the previous psychotherapy experience of most participants. Similarly, among youth with nonsuicidal self-injury, those with experience in dialectical behavioral therapy have suggested incorporating it into a DMHI designed for them [58], showing how previous therapy experience influences preferences for DMHIs. The use of CBT-aligned adaptive coping strategies is important, as research shows that youth with depression use adaptive cognitive strategies less frequently than healthy controls [59] and that applying these strategies is associated with fewer depressive symptoms [60]. Chatbots for youth with depression should incorporate these strategies to create engaging and effective content that aligns with users’ existing coping strategies and effective therapeutic techniques. For example, the chatbot could facilitate social support by helping users identify supportive individuals and suggesting personalized ways to reach out, such as a video call with a friend who is good at cheering them up or a message to a family member who provides good advice. Chatbots can even draft customizable messages to support users who struggle to reach out.

### Attitudes and Expectations Toward Chatbots to Treat Depression

Participants predominantly held positive attitudes toward chatbots to treat depression. Many participants reported feeling less anxious about using a chatbot than seeing a human psychotherapist, especially when discussing sensitive topics. This finding extends research on post-traumatic stress disorder, showing that digital agents lead to greater disclosure of sensitive or stigmatized information [61,62], and supports evidence that youth value the privacy and anonymity offered by DMHIs [63]. Given these findings, chatbots could help users become comfortable with sharing sensitive information and practicing therapeutic conversations, with potential benefits for subsequent sessions with psychotherapists. However, some participants were skeptical about the chatbots’ ability to address individual problems and provide appropriate advice on sensitive topics, which is supported by evidence that commercial chatbots for adults with depression often fail to match user inputs, understand messages, or respond appropriately [64]. Although privacy and data security are frequently cited as primary risks of DMHIs for young people [65], few participants raised these concerns. Instead, they viewed chatbots as privacy-enhancing compared with human therapists. Nevertheless, robust privacy and data security remain essential, and it is unclear whether participants were genuinely unconcerned or simply assumed chatbots would have strong protections in place.

### Design Preferences

Our study revealed important insights into design preferences for chatbots for youth with depression. First, participants expressed diverse preferences for dialogue topics, with three areas standing out: (1) chatting about daily life, (2) discussing urgent problems and receiving advice, and (3) working through therapy exercises that address specific problems. Interestingly, while youth with nonsuicidal self-injury [58], emotional problems [66], and from the general population [63] included being connected to others facing similar challenges as a key feature of their DMHI, our participants ranked such social connection as their lowest priority, highlighting how design preferences may vary across mental health conditions. In summary, a chatbot to treat depression in youth should reflect these preferences. However, available applications have not implemented chats about daily life, and relied on rule-based approaches for problem-solving [14,15], likely due to insufficient conversational capabilities. Structured therapy techniques, such as behavioral activation or cognitive restructuring, are standard components of DMHIs [38] and chatbots [67]. However, their current implementation remains predominantly static [68], without personalized advice or feedback during therapeutic exercises. Large language models (LLMs) such as OpenAI’s GPT or Anthropic’s Claude promise to overcome current limitations of DMHIs and chatbots. LLMs enable chatbots to better address our participants’ design preferences, such as natural conversations about daily life and advice on urgent problems. In addition, LLMs can enhance static implementations of therapeutic exercises by offering personalized guidance and feedback. In behavioral activation, LLMs can personalize the explanation of the relationship between behavior and feelings and evaluate the proposed activity plan for feasibility and therapeutic appropriateness. In cognitive restructuring, Sharma et al [69] demonstrated that LLMs can assist users in identifying thinking traps and generating reframed thoughts. Although these enhanced capabilities have the potential to improve understanding and skill development, key challenges remain. LLMs can generate false or harmful messages [70], posing risks to vulnerable users. Future research needs to explore how capitalize on their advanced conversational abilities while ensuring therapeutic quality and safety [71].

Second, our findings show that engaging and effective chatbots for youth with depression need personalization beyond the conversational capabilities of LLMs. Drawing on the framework by Cohen et al [72] for psychotherapy personalization, effective chatbots require personalization in content selection and interaction style. Content selection ensures that chatbots address the diverse problems and dialogue topics our participants reported, aligning with findings from Li et al [53] and Ludlow et al [66]. A personalized interaction style accommodates the preferences for different social roles and language use. While the benefits of personalizing the chatbot persona have been shown [73,74]. LLMs enable the personalization of interaction styles via prompt instructions, reducing the need to craft different responses for each style manually [75]. Despite the clear need for personalization, participants disagreed on who should control it [76], preventing a clear design recommendation. While some preferred user-led personalization, aligning with Kenny et al [63], others favored chatbot-led personalization or a hybrid approach. Given this ambiguity and the lack of empirical evidence [77], future research is needed to guide chatbot personalization.

### Limitations

Our study has 2 main limitations. First, our sample size was limited, and the participants were predominantly women. Although depression is more prevalent in women [78-80], the absence of men limits the generalizability of our findings across genders. The gender imbalance resulted from convenience sampling through a single youth psychiatrist. One man was informed about the study but declined to participate. While our study included 2 nonbinary participants, an underrepresented demographic in research, recruiting men would have required targeted efforts beyond our study’s constraints. In addition, our sample consisted entirely of participants who had actively sought mental health support, most of whom had previous psychotherapy experience. As a result, perspectives from those who avoid professional help or resist treatment may be underrepresented. Due to these sampling limitations, we likely did not achieve full data saturation. Although qualitative research can reach saturation with as few as twelve interviews [31], our sample composition suggests that some perspectives may have been missed. However, our findings on youth depression and related problems replicated findings from studies with much larger sample sizes [81], indicating that our results are comprehensive. Future studies should recruit a more diverse sample, specifically including men, individuals reluctant to seek professional treatment, and those without previous psychotherapy experience, to validate and extend our findings.

Second, our study captured attitudes, preferences, and hypothetical usage scenarios rather than actual usage behavior. While participants interacted with a prototype during the think-aloud sessions, this brief, controlled interaction may not reflect real-world interactions. The gap between stated preferences and actual behavior is well-documented [82], and our study design does not allow us to determine whether the design preferences of the participants would translate into long-term engagement or effective use. Future research should follow the next steps of an iterative human-centered design process [83,84] with functional prototypes to assess whether the identified design preferences lead to actual user engagement and therapeutic benefits. Despite these limitations, our study provides in-depth findings on the design of chatbots to treat depression in youth, highlighting the value of qualitative research in the iterative development of DMHIs [83,84].

### Conclusions and Future Work

Our study provides valuable insights into the problems and coping strategies of youth with depression, and their attitudes, expectations, and design preferences for a chatbot to treat depression in youth. We found complex user needs, predominantly positive attitudes toward chatbots, and various design preferences, including the need for diverse dialogue topics and personalization. Our findings led to concrete design recommendations that lay a crucial foundation for developing engaging and effective chatbots to treat depression in youth.

Despite these contributions, several research directions remain. First, future studies should validate and extend these findings with larger, more diverse samples to ensure broader representation of youth with depression. Second, examining actual usage patterns and long-term engagement with functional chatbot prototypes will help assess the effectiveness of our design recommendations. Third, investigating the responsible integration of LLMs is important, including the development of robust safeguards and evaluating dialogue quality and therapeutic outcomes. Finally, researchers must design and evaluate effective personalization features, particularly regarding whether users, the chatbot or both should control personalization, while balancing implementation efforts and impact. By addressing these research directions, we can further improve chatbots to treat depression in youth and ultimately contribute to more accessible, engaging, and effective mental health support for this vulnerable population.

## Supplementary material

10.2196/66632 Multimedia Appendix 1 Interview Guide.

10.2196/66632 Multimedia Appendix 2 Categories.

10.2196/66632 Multimedia Appendix 3 Informed written consent.

## Abbreviations

CBT: cognitive behavioral therapy
DMHI: digital mental health intervention
DSM-5: *Diagnostic and Statistical Manual of Mental Disorders, Fifth Edition*
LLM: large language model
PHQ-8: 8-item Patient Health Questionnaire
SCARED: Screen for Child Anxiety Related Disorders

## References

[1] Clayborne ZM, Varin M, Colman I (2019). Systematic review and meta-analysis: adolescent depression and long-term psychosocial outcomes. J Am Acad Child Adolesc Psychiatry.

[2] Thapar A, Collishaw S, Pine DS, Thapar AK (2012). Depression in adolescence. Lancet.

[3] Oud M, de Winter L, Vermeulen-Smit E (2019). Effectiveness of CBT for children and adolescents with depression: a systematic review and meta-regression analysis. Eur Psychiatry.

[4] Eisenberg D, Golberstein E, Gollust SE (2007). Help-seeking and access to mental health care in a university student population. Med Care.

[5] Ebert DD, Mortier P, Kaehlke F (2019). Barriers of mental health treatment utilization among first-year college students: first cross-national results from the WHO World Mental Health International College student initiative. Int J Methods Psychiatr Res.

[6] Gulliver A, Griffiths KM, Christensen H (2010). Perceived barriers and facilitators to mental health help-seeking in young people: a systematic review. BMC Psychiatry.

[7] Radez J, Reardon T, Creswell C, Lawrence PJ, Evdoka-Burton G, Waite P (2021). Why do children and adolescents (not) seek and access professional help for their mental health problems? A systematic review of quantitative and qualitative studies. Eur Child Adolesc Psychiatry.

[8] Domhan D, In-Albon T, Pfeiffer S (2023). Erfassung von Barrieren und Faszilitatoren zur Aufnahme einer Psychotherapie im Kontext ambulanter Kinder- und Jugendlichenpsychotherapie. Psychotherapie.

[9] Wu Y, Fenfen E, Wang Y (2023). Efficacy of internet-based cognitive-behavioral therapy for depression in adolescents: a systematic review and meta-analysis. Internet Interv.

[10] Leech T, Dorstyn D, Taylor A, Li W (2021). Mental health apps for adolescents and young adults: a systematic review of randomised controlled trials. Child Youth Serv Rev.

[11] Cameron SK, Rodgers J, Dagnan D (2018). The relationship between the therapeutic alliance and clinical outcomes in cognitive behaviour therapy for adults with depression: a meta‐analytic review. Clin Psychology and Psychoth.

[12] Vaidyam AN, Wisniewski H, Halamka JD, Kashavan MS, Torous JB (2019). Chatbots and conversational agents in mental health: a review of the psychiatric landscape. Can J Psychiatry.

[13] Bendig E, Erb B, Schulze-Thuesing L, Baumeister H (2019). Die nächste generation: chatbots in der klinischen psychologie und psychotherapie zur förderung mentaler gesundheit – ein scoping-review. Verhaltenstherapie.

[14] Inkster B, Sarda S, Subramanian V (2018). An empathy-driven, conversational artificial intelligence agent (Wysa) for digital mental well-being: real-world data evaluation mixed-methods study. JMIR Mhealth Uhealth.

[15] Fitzpatrick KK, Darcy A, Vierhile M (2017). Delivering cognitive behavior therapy to young adults with symptoms of depression and anxiety using a fully automated conversational agent (Woebot): a randomized controlled trial. JMIR Ment Health.

[16] Abd-Alrazaq AA, Rababeh A, Alajlani M, Bewick BM, Househ M (2020). Effectiveness and safety of using chatbots to improve mental health: systematic review and meta-analysis. J Med Internet Res.

[17] Perski O, Crane D, Beard E, Brown J (2019). Does the addition of a supportive chatbot promote user engagement with a smoking cessation app? An experimental study. Digit Health.

[18] Linardon J, Torous J, Firth J, Cuijpers P, Messer M, Fuller-Tyszkiewicz M (2024). Current evidence on the efficacy of mental health smartphone apps for symptoms of depression and anxiety. A meta-analysis of 176 randomized controlled trials. World Psychiatry.

[19] Darcy A, Daniels J, Salinger D, Wicks P, Robinson A (2021). Evidence of human-level bonds established with a digital conversational agent: cross-sectional, retrospective observational study. JMIR Form Res.

[20] Bae Brandtzæg PB, Skjuve M, Kristoffer Dysthe KK, Følstad A When the social becomes non-human: young people’s perception of social support in chatbots.

[21] Beatty C, Malik T, Meheli S, Sinha C (2022). Evaluating the therapeutic alliance with a free-text CBT conversational agent (Wysa): a mixed-methods study. Front Digit Health.

[22] Feine J, Gnewuch U, Morana S, Maedche A (2019). A taxonomy of social cues for conversational agents. Int J Hum Comput Stud.

[23] Vaidyam AN, Linggonegoro D, Torous J (2021). Changes to the psychiatric chatbot landscape: a systematic review of conversational agents in serious mental illness: changements du paysage psychiatrique des chatbots: une revue systématique des agents conversationnels dans la maladie mentale sérieuse. Can J Psychiatry.

[24] Cicchetti D, Toth SL (2009). A Developmental Psychopathology Perspective on Adolescent Depression Handbook of Depression in Adolescents.

[25] Rice F, Riglin L, Lomax T (2019). Adolescent and adult differences in major depression symptom profiles. J Affect Disord.

[26] Andone I, Błaszkiewicz K, Eibes M, Trendafilov B, Montag C, Markowetz A How age and gender affect smartphone usage.

[27] Huffman S OMG! Mobile voice survey reveals teens love to talk.

[28] Agapie E, Chang K, Patrachari S, Neary M, Schueller SM (2022). Understanding mental health apps for youth: focus group study with Latinx youth. JMIR Form Res.

[29] Høiland CG, Følstad A, Karahasanovic A (2020). Hi, can I help? Exploring how to design a mental health chatbot for youths. Human Technology.

[30] Grové C (2020). Co-developing a mental health and wellbeing chatbot with and for young people. Front Psychiatry.

[31] Guest G, Bunce A, Johnson L (2006). How many interviews are enough? An experiment with data saturation and variability. Field Methods US: Sage Publications.

[32] Association AP (2013). Diagnostic and Statistical Manual of Mental Disorders (DSM-5®).

[33] Kaufman J, Birmaher B, Brent D (1997). Schedule for Affective Disorders and Schizophrenia for School-Age Children-Present and Lifetime Version (K-SADS-PL): initial reliability and validity data. J Am Acad Child Adolesc Psychiatry.

[34] Martin A, Rief W, Klaiberg A, Braehler E (2006). Validity of the Brief Patient Health Questionnaire Mood Scale (PHQ-9) in the general population. Gen Hosp Psychiatry.

[35] Weitkamp K, Romer G, Rosenthal S, Wiegand-Grefe S, Daniels J (2010). German Screen for Child Anxiety Related Emotional Disorders (SCARED): reliability, validity, and cross-informant agreement in a clinical sample. Child Adolesc Psychiatry Ment Health.

[36] Towery J (2016). The Anti-Depressant Book: A Practical Guide for Teens and Young Adults to Overcome Depression and Stay Healthy.

[37] Auerbach RP, Webb CA, Stewart JG (2016). Cognitive Behavior Therapy for Depressed Adolescents: A Practical Guide to Management and Treatment.

[38] Huguet A, Rao S, McGrath PJ (2016). A systematic review of cognitive behavioral therapy and behavioral activation apps for depression. PLOS ONE.

[39] Lejuez CW, Hopko DR, Hopko SD (2001). A brief behavioral activation treatment for depression. Treatment manual. Behav Modif.

[40] Abel U, Hautzinger M (2013). Kognitive Verhaltenstherapie Bei Depressionen Im Kindes- Und Jugendalter.

[41] Groen G, Petermann F (2015). Therapie Tools Depression Im Kindes-Und Jugendalter.

[42] Botsociety Design, preview and prototype your next chatbot or voice assistant. https://botsociety.io.

[43] Jaspers MWM, Steen T, van den Bos C, Geenen M (2004). The think aloud method: a guide to user interface design. Int J Med Inform.

[44] Mayring P, Fenzl T, Baur N, Blasius J (2019). Handbuch Methoden Der Empirischen Sozialforschung.

[45] Mayring P (2015). Qualitative Inhaltsanalyse: Grundlagen Und Techniken 12, Überarbeitete Auflage.

[46] Fenzl T, Mayring P (2017). QCAmap: eine interaktive webapplikation für qualitative inhaltsanalyse. ZPID (Leibniz Institute for Psychology Information).

[47] Zehetmair C, Nagy E, Leetz C (2020). Self-practice of stabilizing and guided imagery techniques for traumatized refugees via digital audio files: qualitative study. J Med Internet Res.

[48] Kroenke K, Strine TW, Spitzer RL, Williams JBW, Berry JT, Mokdad AH (2009). The PHQ-8 as a measure of current depression in the general population. J Affect Disord.

[49] Caporino NE, Sakolsky D, Brodman DM (2017). Establishing clinical cutoffs for response and remission on the Screen for Child Anxiety Related Emotional Disorders (SCARED). J Am Acad Child Adolesc Psychiatry.

[50] American Psychiatric Association (2013). 5th.

[51] Agnafors S, Norman Kjellström A, Torgerson J, Rusner M (2019). Somatic comorbidity in children and adolescents with psychiatric disorders. Eur Child Adolesc Psychiatry.

[52] Mullarkey MC, Marchetti I, Beevers CG (2019). Using network analysis to identify central symptoms of adolescent depression. J Clin Child Adolesc Psychol.

[53] Li SH, Achilles MR, Spanos S, Habak S, Werner-Seidler A, O’Dea B (2022). A cognitive behavioural therapy smartphone app for adolescent depression and anxiety: co-design of ClearlyMe. tCBT.

[54] Radez J, Reardon T, Creswell C, Orchard F, Waite P (2022). Adolescents’ perceived barriers and facilitators to seeking and accessing professional help for anxiety and depressive disorders: a qualitative interview study. Eur Child Adolesc Psychiatry.

[55] Cuijpers P, Karyotaki E, Ciharova M (2023). The effects of psychological treatments of depression in children and adolescents on response, reliable change, and deterioration: a systematic review and meta-analysis. Eur Child Adolesc Psychiatry.

[56] Wenzel A (2017). Basic strategies of cognitive behavioral therapy. Psychiatr Clin North Am.

[57] Luxton R, Kyriakopoulos M (2020). Depression in children and young people: identification and management NICE guidelines. Arch Dis Child Educ Pract Ed.

[58] Čuš A, Edbrooke-Childs J, Ohmann S, Plener PL, Akkaya-Kalayci T (2021). “Smartphone apps are cool, but do they help me?”: a qualitative interview study of adolescents’ perspectives on using smartphone interventions to manage nonsuicidal self-injury. Int J Environ Res Public Health.

[59] Mihailescu I, Efrim-Budisteanu M, Andrei LE (2023). Cognitive coping strategies among inpatient adolescents with depression and psychiatric comorbidity. Children (Basel).

[60] Schäfer JÖ, Naumann E, Holmes EA, Tuschen-Caffier B, Samson AC (2017). Emotion regulation strategies in depressive and anxiety symptoms in youth: a meta-analytic review. J Youth Adolescence.

[61] Lucas GM, Rizzo A, Gratch J (2017). Reporting mental health symptoms: breaking down barriers to care with virtual human interviewers. Front Robot AI.

[62] Pickard MD, Roster CA, Chen Y (2016). Revealing sensitive information in personal interviews: Is self-disclosure easier with humans or avatars and under what conditions?. Comput Human Behav.

[63] Kenny R, Dooley B, Fitzgerald A (2016). Developing mental health mobile apps: exploring adolescents’ perspectives. Health Informatics J.

[64] Martinengo L, Lum E, Car J (2022). Evaluation of chatbot-delivered interventions for self-management of depression: content analysis. J Affect Disord.

[65] Wies B, Landers C, Ienca M (2021). Digital mental health for young people: a scoping review of ethical promises and challenges. Front Digit Health.

[66] Ludlow K, Russell JK, Ryan B (2023). Co-designing a digital mental health platform, “Momentum”, with young people aged 7-17: a qualitative study. Digit Health.

[67] Ahmed A, Hassan A, Aziz S (2023). Chatbot features for anxiety and depression: a scoping review. Health Informatics J.

[68] Denecke K, Schmid N, Nüssli S (2022). Implementation of cognitive behavioral therapy in e-mental health apps: literature review. J Med Internet Res.

[69] Sharma A, Rushton K, Lin IW, Nguyen T, Althoff T (2024). Facilitating self-guided mental health interventions through human-language model interaction: a case study of cognitive restructuring. https://dl.acm.org/doi/proceedings/10.1145/3613904.

[70] Birkun AA, Gautam A (2023). Large language model (LLM)-powered chatbots fail to generate guideline-consistent content on resuscitation and may provide potentially harmful advice. Prehosp Disaster Med.

[71] Stade EC, Stirman SW, Ungar LH (2024). Large language models could change the future of behavioral healthcare: a proposal for responsible development and evaluation. Npj Ment Health Res.

[72] Cohen ZD, Delgadillo J, DeRubeis RJ (2021). Bergin and Garfield’s Handbook of Psychotherapy and Behavior Change.

[73] Nißen M, Rüegger D, Stieger M (2022). The effects of health care chatbot personas with different social roles on the client-chatbot bond and usage intentions: development of a design codebook and web-based study. J Med Internet Res.

[74] Ahmad R, Siemon D, Gnewuch U, Robra-Bissantz S (2022). Designing personality-adaptive conversational agents for mental health care. Inf Syst Front.

[75] Jiang H, Zhang X, Cao X, Breazeal C, Roy D, Kabbara J PersonaLLM: investigating the ability of large language models to express personality traits. https://aclanthology.org/2024.findings-naacl.

[76] Fan H, Poole MS (2006). What is personalization? Perspectives on the design and implementation of personalization in information systems. JOCEC.

[77] Kocaballi AB, Berkovsky S, Quiroz JC (2019). The personalization of conversational agents in health care: systematic review. J Med Internet Res.

[78] Avenevoli S, Swendsen J, He JP, Burstein M, Merikangas KR (2015). Major depression in the national comorbidity survey-adolescent supplement: prevalence, correlates, and treatment. J Am Acad Child Adolesc Psychiatry.

[79] Seedat S, Scott KM, Angermeyer MC (2009). Cross-national associations between gender and mental disorders in the World Health Organization World Mental Health Surveys. Arch Gen Psychiatry.

[80] Hua Z, Wang S, Yuan X (2024). Trends in age-standardized incidence rates of depression in adolescents aged 10-24 in 204 countries and regions from 1990 to 2019. J Affect Disord.

[81] Chevance A, Ravaud P, Tomlinson A (2020). Identifying outcomes for depression that matter to patients, informal caregivers, and health-care professionals: qualitative content analysis of a large international online survey. Lancet Psychiatry.

[82] de Corte K, Cairns J, Grieve R (2021). Stated versus revealed preferences: an approach to reduce bias. Health Econ.

[83] Yardley L, Morrison L, Bradbury K, Muller I (2015). The person-based approach to intervention development: application to digital health-related behavior change interventions. J Med Internet Res.

[84] Harte R, Glynn L, Rodríguez-Molinero A (2017). A human-centered design methodology to enhance the usability, human factors, and user experience of connected health systems: a three-phase methodology. JMIR Hum Factors.

