# Supplementary material for: Designing Chatbots to Treat Depression in Youth: Qualitative Study
Source: JMIR Hum Factors. 2025 Jun 19;12:e66632. doi: 10.2196/66632 (PMC12199846; doi:10.2196/66632)
Supplement: Multimedia Appendix 1 [file humanfactors-v12-e66632-s001.docx]

## Multimedia Appendix 1

### Interview Guide (translated from German)

1. **Warm-Up**

You've already answered a few questions.

Thank you again for taking part in our study. We depend on young people like you. You are experts on your experiences and problems. That's why I want to talk to you about a few things in more detail in the interview. Here too, there is no right or wrong, but I want to know what you think. If you find a question strange or don't feel you understand something, please let me know.

Do you still have any questions?

If not, I'll start the recording, and we'll get started with the questions. But you can always ask questions in between.

1. **Interview Questions**

**Note**: The main questions are highlighted in **bold**, whereas potential follow-up questions are displayed as lists below the main question.

| **Introductory question** | **Why did you decide to take part in our study?**  **If you weren't here, what would you be doing right now?** |
| --- | --- |
| **1. Topic:**  **Problems & coping strategies** | **How do you feel when you're not doing so well?**   - What situations do you find difficult then? - How do you feel then? - How do you behave then? - What thoughts do you have then? - What stresses you out the most? - What do you need help with then? |
|  | **What have you tried in the past to make you feel better?**   - What has helped you and what hasn't? - Where have you gone for help/information? - What stopped you from getting help? - How did you end up getting help after all? - How could the help have been even better for you personally? - What would you hope for/not want if you were to get help? - How do you know that something has helped you? - How do you know that you are no longer depressed? |
| **2. Thema: Chatbots** | **What do you imagine when you think of psychotherapy?**  **Have you had any experience with psychotherapy?**   - Explanation if you have a very clichéd idea or no idea at all: In psychotherapy, the therapist helps you with your problems. This means that you work together to find out how you can get better. To do this, you first get to know each other and then the therapist has lots of strategies and tools that you can try out together. |
|  | **Do you know what a chatbot is?**   - Imagine Siri or Alexa can not only talk to you, but also write to you. - Imagine you are writing to someone on WhatsApp, but not to a real person, but to a computer/robot |
|  | **What do you think it would be like for you to use a chatbot that is there to help you with <previously mentioned problems>?**   - What do you think it would be like for you to use a chatbot that is there to help you with <previously mentioned problems>? - What could be good? (positive expectations) - What concerns do you have? (negative expectations) - How could your <concerns> be minimised? - Do you think that this could make you feel better? (Expectation of success?) - How would people around you react to this? - What would motivate you to use it? |
|  | **How do you imagine an ideal conversation with the chatbot?**   - What should the chatbot be able to do? - What topics would you talk about? - What questions should the chatbot ask? - Possibilities: Talk about your everyday life and your problems, Do therapeutic exercises to work on one of your problems (e.g. <sleep>) - How should the chatbot behave? - What personality should the chatbot have? (Personality / Role) - More like a friend your age, like a therapist or like a trainer/mentor? (Goal: Name personality traits) - What adjectives would you use to describe your perfect chatbot? - Option: Several personalities, depending on what you need at the moment (e.g. Friendly for everyday worries, Therapeutic for exercises) - Who should start the conversation? - Rather yourself or the chatbot starts, among other things with reminders and messages? |
|  | **Imagine you are thinking about getting such a chatbot. How would the chatbot have to be implemented so that you would download and use it?**   - Writing/chatting (WhatsApp) vs. speaking (Siri, Alexa)? - When chatting: Only text messages or also voice messages, pictures, videos (therapy content), memes, gifs, emojis? - Medium through which it is played : Would you rather use your own app or chat in a messenger app (e.g. WhatsApp) as with a human contact? - If your own app: How should your own app be designed? - Personalization   - Do you want to personalize the chatbot, i.e. tailor it to you? What would you like to personalize/customize?   - e.g. tailor the topics of conversation or exercises to you, choose the avatar and personality of the bot, set your own avatar, give the bot a name.   - Do you want to make these settings yourself or should the chatbot automatically adjust to you? - Time   - How long should a conversation/exercise last?   - How many times a week would you want to use the chatbot?   - How much time do you have per week/day to use such a chatbot?   - For how many days/weeks do you want to use the chatbot? - Intelligence   - would you rather write texts yourself or click on suggested answers?   - should the chatbot remember things about you, i.e. get to know me? |
| **Abschlussfrage** | **Is there anything else you would like to add about chatbots?**  **Would you be happy for us to save your contact details for future studies and get back to you if necessary?**  **Thank the person for participating.** |

Further Questions:

- Can you give me an example?
- In which situation have you experienced this?
- What happened then?
- What did you expect?
- What do you mean when you say "..."?
- Wait and see!
- 5 consecutive why-questions (if you have difficulty putting something into words)
