# Supplementary material for: Designing Chatbots to Treat Depression in Youth: Qualitative Study
Source: JMIR Hum Factors. 2025 Jun 19;12:e66632. doi: 10.2196/66632 (PMC12199846; doi:10.2196/66632)
Supplement: Multimedia Appendix 2 [file humanfactors-v12-e66632-s002.docx]

## Multimedia Appendix 2

Table S2. Problems.

| **Main Category** | **Subcategories** | **N** | **%** |
| --- | --- | --- | --- |
| Depressive symptoms | - lack of motivation and listlessness - depressed mood and less enjoyment of life - self-doubt, self-criticism, self-devaluation - difficulty coping with everyday tasks or meeting basic needs - difficulty eating - difficulty getting up - irritable towards others, difficulty being nice to others - helpless in the face of depressive symptoms - ruminating - pessimistic attitude - feeling lonely and alone - feeling of meaninglessness - impairment due to intense feelings or stress - feeling of emptiness - sleep problems - no longer pursue familiar and fun activities - self-harm - devaluation of one's own appearance - suicidality - difficulty concentrating - guilt - boredom - severe exhaustion - weight gain due to antidepressants - mood swings - feeling nervous | 14 | 100 |
| Interpersonal problems | - social withdrawal - stress caused by parents - friendship breaks - don't want to burden anyone - relationship breakdowns as a stressor - inhibition to be open, with friends who "know you too well" - bullying - parental violence - social conflicts over food - more difficult to confide in the family than with friends | 14 | 100 |
| Worries and problems regarding school and professional future | - worries about one's own future - pressure - negative experiences at school, school absenteeism - concerns and pressures about planning for the future | 11 | 78 |
| Barriers for seeking help | - fear of talking about problems and being judged - difficulty getting professional help - seeing your own problems as "not bad enough" - big barriers to seek professional support - double standards: recommending help to others rather than oneself - fear of feedback that one's own problems are not "bad enough" - don’t try out help offers, as places are scarce - not daring to seek help - no ideas what could help | 12 | 85 |
| Comorbidities | - social anxiety - panic attacks - fears - alcohol consumption - compulsive behavior | 9 | 64 |
| Problems in therapy | - negative experiences in healthcare systems - strategies/offers of help only experienced as helpful in the short term or not at all - difficulty opening up or trusting - pressure to perform and failure in therapy - breach of trust in therapeutic contexts - forgetting familiar coping strategies - discontinuation of therapy in good phases - too much detachment from therapists - large age difference to therapists - being treated from above | 9 | 64 |
| Stigma | - adults stigmatize or trivialize the problems - feeling different from others - others don’t understand the problems - symptoms of illness are evaluated negatively (e.g. "crazy") | 6 | 42 |
| Physical Problems | - tension - somatic complaints | 4 | 28 |

Table S3. Coping Strategies.

| **Category** | **Subcategories** | **N** | **%** |
| --- | --- | --- | --- |
| Social support | - from specific people: friends, family, partners, teachers or people online - talking to someone to get it off the chest - communicating that you are not feeling well and what you currently need | 14 | 100 |
| Distractions and positive activities | - going outside - media consumption - doing something fun - sleeping - playing with animals - painting - listening to music - seeking distance to everyday surroundings and people - waiting for it to be over - self-harm - listening to loud music - talking about beautiful things and coming up with other thoughts - appreciating things - dancing | 13 | 92 |
| Professional care | - from therapist - from doctor | 9 | 64 |
| Cognitive strategies | - talking to yourself well - writing (e.g. diary) - self-reflection - suppressing or covering up tension - humor and self-irony - less ruminating - taking pressure from yourself - building self-esteem - countering negative thoughts with positive thoughts - expressing feelings - acknowledging problems as a disease - describing problems and feelings | 9 | 64 |
| Structure | - establish structure and habits - sorting yourself and your thoughts - getting up early - creating a list of activities for the day - setting goals - making plans - taking small steps and focusing on small successes - making rules for yourself - focusing on acute problems - consciously breaking out of routines | 6 | 42 |
| Focus on your needs | - actively taking time for yourself - leaving or avoiding stressful social situations - withdrawing - paying attention to basic needs - distancing yourself from things that are not good for you - focusing on yourself | 7 | 50 |
| Mindfulness | - breathing techniques - meditation - mindfulness exercises | 3 | 21 |
| Psychoeducation | - online - trying to understand symptoms | 2 | 14 |

Table S4. Attitudes and Expectations.

| **Main Category** | **Subcategory** | **N** | **%** |
| --- | --- | --- | --- |
| More personal | - permanent contact person, not feeling left alone - more human (than other apps) - higher motivation to use it (than other apps) - more personal (than other apps) | 3 | 21 |
| Less anxiety about therapy | - Contact person for topics you can't talk about with anyone else or when no one else is there - neutral opinion, no fear of negative reactions - feel comfortable - easier than with a real person - easier to be open when writing compared to speaking - Social norms/ lower pressure - No feelings of guilt because you don’t burden anyone - It can't get too much, chatbot will always be there | 12 | 85 |
| Expectations of improvement | - helpful for most problems and topics - everyday tool - writing down thoughts helpful - assist in researching problems and symptoms | 8 | 57 |
| Interested to try |  | 9 | 64 |
| Unlimited capacity and flexibility | - no waiting times - unlimited use - available at any time - gives hope - more low-threshold than looking for a therapist or going to therapy - good alternative if no therapist is available | 11 | 78 |
| Low intrinsic drive | - lack of motivation to use it because of depressive symptoms - lack of social pressure compared to therapy - forget about it in relevant moments - self-help requires some initiative | 2 | 14 |
| Concerns about data security |  | 2 | 14 |
| Concerns about chatbot’s intelligence / natural language capabilities | - fear of being disappointed - will not be able to address individual, diverse or unusual problems - difficult to balance personal and professional style - inappropriate answers to emotional and intimate topics or inappropriate suggestions to problems - will not be able to know what is needed at the moment, e.g. emotion-focused or solution-oriented support | 10 | 71 |
| Concerns about how the conversations will feel | - won’t feel like a conversation with a real person - too robotic, cold or analytical - too human would be creepy - more effortful than talking with a human therapist | 9 | 64 |

Table S5. Design Preferences.

| **Main Category** | **Subcategory** | **Subcategory** | **N** | **%** |
| --- | --- | --- | --- | --- |
| **Personalization** | Elements to personalize | - mobile app: profile picture, notifications, username, color theme - chatbot: personality, gender, media use (videos, GIFs, emojis), avatar - dialogue topics / content | 13 | 92 |
|  | Control over personalization | - system / chatbot - user - hybrid: system / chatbot and user | 12 | 85 |
|  | Timing | - static: one time at first use - dynamically | 11 | 78 |
|  | Concerns | - personalizing content /dialogue topics could provoke avoidance behavior - personalization could reduce the seriousness | 2 | 14 |
| **Data Security and Privacy** |  | - data security and privacy crucial - anonymous use should be possible - write terms and conditions in a comprehensible and accessible manner - user should decide which data is (not) saved - privacy concerns if integrated into messaging app like WhatsApp | 4 | 28 |
| **Dialogue Topics / Content** |  | - therapeutic exercises - reminders for basic needs - chatbot should support user to confide in other people - emotion regulation - tackling recurring thoughts - distractions - discuss current problems and suggest solutions - cover daily life - assessment / diagnostics - psychoeducation | 14 | 100 |
| **Personality** |  | - understanding/ sensitive - friendly/ kind - empathic - share values of the user (e.g. not homophobic) - encouraging - reliable - caring - interested - good understanding of human nature - motivating/ uplifting - open and tolerant - personal - not too stiff, but appropriate for young people - talkative - solidary - neutral/objective, - non-judgmental - no youth slang - clear tone and not too emotional - human-like - humorous | 14 | 100 |
| **Social Role** |  | - friend - therapist - a mixture of friend and therapist | 14 | 100 |
| **User Interface** | Input modalities | - a mix of pre-defined answers and free text input - text - text and voice input | 13 | 92 |
|  | Standalone app | - rather a standalone app than a messenger integration | 12 | 85 |
|  | Appealing user interface design | - appealing - bright colors - mix of colorful and plain design - age-appropriate - light mode - dark mode - clear - simple, minimalistic | 13 | 92 |
|  | User friendly and trustworthy | - quickly usable, brief introduction - easy to use - content created by experts - trustworthy | 5 | 35 |
|  | Features | - emergency mode, e.g. include emergency numbers - support for seeking professional, human help - main menu and settings menu - save insights (such as diagnoses or strategies) in the app, outside the chat - notifications | 14 | 100 |
