# Supplementary material for: Designing Chatbots to Treat Depression in Youth: Qualitative Study
Source: JMIR Hum Factors. 2025 Jun 19;12:e66632. doi: 10.2196/66632 (PMC12199846; doi:10.2196/66632)
Supplement: Multimedia Appendix 3 [file humanfactors-v12-e66632-s003.pdf]

EBERHARD KARLS UNIVERSITÄT TÜBINGEN

Mathematical and scientific Faculty

Department of Psychology
Clinical Psychology and Psychotherapy

Dipl.-Psych. Stefan Lüttke
Schleichstr. 4, 72076 Tübingen
Phone: +49 7071 29-74358
E-mail: stefan.luettke@uni-tuebingen.de

Important note: Your parents will also receive a declaration of consent from us and please sign it as well.

Please bring the signed consent forms (yours and those of your parents) with you to the examination appointment.

Please keep a copy of your consent form to yourself or give it to your parents.

Declaration of consent for young people

Version: 1.0

Title of the study: Conversational agent for the treatment of depression in youth (CADY) – patient-centered needs assessment

[German title of the study: Chatbot for the treatment of depression in adolescents - patient-centered needs analysis]

I

____

(Your name in BLOCK LETTERS)

I have been informed in writing about the above-mentioned study and the experimental procedure. I consent to my data being collected and processed as described in the study information.

If I had additional questions about this study, they were answered by Mr./Mrs.

____

(only if applicable: name of the study assistant in BLOCK FONT who answered your questions)

answered completely and to my satisfaction.

I agree that the following data will be collected and processed by me:

(1) Data needed to organize your studies:

My declaration of consent and contact details

(2) Study data:
My age, gender, mother tongue, education, owning a smartphone; whether my "thinking skills" are sufficient for participation in the study; whether I'm currently feeling so bad that I think it would be better to be dead; whether there is currently a psychosis (e.g. that I hear voices that do not exist at all); whether I have mental disorders (current and/or past); who has studied these mental disorders; whether I am currently taking medication for the treatment of mental disorders (currently); whether I have needed psychotherapeutic help (currently and/or in the past);

Whether I currently have depressive symptoms and how severe they are; whether I currently have fears and how strong they are; what characteristics the psychotherapy chatbot should have for the treatment of depressive symptoms;

My statements in the chatbot interview on the following topics: What problems depression caused me or has caused me in the past and what solutions I have or had for it; what advantages and disadvantages I see in a psychotherapy chatbot to treat depression; how I would design such a psychotherapy chatbot.

My study data will be stored with a pseudonym in the Department of Psychology at the University of Tübingen. This means that my data is stored with a number and my name is not mentioned.

The chatbot interview is recorded with a digital recording device for sound recordings. My voice in the audio recording is alienated immediately after the examination appointment and the corresponding audio file is stored encrypted and pseudonymised.

There is a coding list on paper that connects my name to this number. This coding list is only accessible to the experimenter and the project manager, which means that only these people can associate my data with my name.

After the data has been collected from all study participants, the coding list and my contact details will be deleted. This will happen by 31.08.2022 at the latest. My data is then anonymized. This means that it is no longer possible for anyone to associate my data with my name.

I know that I can revoke my consent to the storage of my data without any disadvantages for me. I can request the deletion of my data at any time. However, if the encoding list has already been deleted, it is no longer possible to find out which data belongs to me. The data can then no longer be deleted, as it is anonymized.

I agree that my fully anonymized data can be used for research. For this purpose, the data is stored in a research data center for an unlimited period of time. The research data center is called "PsychData" and is located at the Leibniz Institute for Psychology (ZPID) in Trier, Germany. Only scientists are allowed to work with the completely anonymized data.

I agree that I will receive an expense allowance of EUR 30.00 in cash and against signing a receipt for my participation in the study.

I have had enough time to decide to participate in the study and agree to participate in the study. I know that my participation in the study is voluntary. I know that I can end my participation in the study at any time without giving reasons. I know that in this case I will receive an expense allowance for the time spent until the abortion.

I have received a copy of the study information and a copy of the informed consent. The information about the study is part of the informed consent form.

Consent to study participation

I agree to participate in the study:

____ ____________________________________

Your surname, first name in **BLOCK FONT** Location, Date

____

Your signature

Consent to the collection and processing of personal data

I agree that personal data may be collected and processed by me as described in the information on the study.

____ ____________________________________

Your surname, first name in **BLOCK FONT** Location, Date

____

Your signature

EBERHARD KARLS UNIVERSITÄT TÜBINGEN

Mathematical and scientific Faculty

Department of Psychology
Clinical Psychology and Psychotherapy

Dipl.-Psych. Stefan Lüttke
Schleichstr. 4, 72076 Tübingen
Phone: +49 7071 29-74358
E-mail: stefan.luettke@uni-tuebingen.de

Important note: Your child will receive a declaration of consent from us in age-appropriate language and please sign it as well.

Please bring the signed declarations of consent with your child to the examination appointment.

Please keep a copy of the respective declarations of consent with your records.

Declaration of consent for legal guardians

Version: 1.0

Title of the study: Conversational agent for the treatment of depression in youth (CADY) – patient-centered needs assessment

[German title of the study: Chatbot for the treatment of depression in adolescents - patient-centered needs analysis]

I, legal guardian of

____

(Name of the participating CHILD in BLOCK LETTERS)

I have been informed in writing about the above-mentioned study and the experimental procedure. I consent to the collection and processing of my data and my child's data as described in the study information.

If I had additional questions about this study, they were answered by Mr./Mrs.

____

(only if applicable: name of the study assistant in BLOCK FONT who answered your questions)

answered completely and to my satisfaction.

I agree to the collection and processing of the following data as described:

(1) Data on the organization of studies (collected from me and my child):
Declaration of consent and contact details

(2) Study data (only collected from my child):
Age; Sex; Mother tongue; School education; Possession of a smartphone; Presence of cognitive abilities that are too low for participation in the study; Presence of suicidal behavior and psychosis (current in each case); Presence of mental disorders (current and/or past); who has been diagnosed with these mental disorders (if applicable); taking medication for the treatment of mental disorders (current); use of psychotherapeutic help (current and/or past); Presence and extent of depressive symptoms and anxiety disorders (current in each case); desired characteristics of a psychotherapy chatbot for the treatment of depressive symptoms; Statements by your child in the chatbot interview on the following topics: Central problems and solution strategies in depression, advantages and disadvantages of a psychotherapy chatbot for the treatment of depressive symptoms, design suggestions for such a psychotherapy chatbot.

The recording of the above-mentioned study data is pseudonymised in the Department of Psychology, Clinical Psychology and Psychotherapy, at the University of Tübingen, using a number and without giving the name of my child.

The chatbot interview is recorded with a digital recording device for sound recordings. My child's voice in the audio recording is alienated immediately after the examination appointment and the corresponding audio file is stored encrypted and pseudonymised.

There is a coding list on paper that connects my child's name to this number. This coding list is only accessible to the experimenter and the project manager, which means that only these persons can associate the collected data with the name of my child.

After completion of the data collection and before the data evaluation, no later than 31.08.2022, the coding list and contact details will be deleted. My child's data is then anonymized. This means that it is no longer possible for anyone to associate the collected data with my child's name.

I am informed that I and my child can revoke my consent to the storage or storage of this data without any disadvantages for me and my child. I and my child can request the deletion of all data at any time. However, if the coding list has already been deleted, my child's record can no longer be identified and therefore no longer deleted. My child's data is then anonymized.

I agree that the completely anonymized data of my child can be used for research purposes. For this purpose, this data is stored in a certified research data center for an unlimited period of time. The research data center is the "PsychData" at the Leibniz Institute for Psychology (ZPID) in Trier, Germany. The fully anonymised data will only be made available to authorised researchers upon request.

I agree that my child will receive an expense allowance of EUR 30.00 in cash and against signing a receipt for his or her participation in the study.

I have had enough time to make a decision and I agree that my child will participate in the above study. I understand that my child's participation in the study is voluntary and that both I and my child can stop participating in the study at any time without giving any reason. I know that in this case I am entitled to an expense allowance for the time spent until the abortion.

I have received a copy of the information on the study for legal guardians and a copy of the declaration of consent. The information on the study for guardians is part of this declaration of consent.

Consent to your child's participation in the study

I consent to my **CHILD's participation** in the study:

____

Surname, first name of the **participating CHILD** in **block letters**

____ ____________________________________

Surname, first name **of the parent or legal guardian** in **BLOCK**

Location, Date

____

Signature of legal guardian

Consent to the collection and processing of personal data

I consent to the collection and processing of personal data of me and my child in the form described in the information on the study.

____

Surname, first name of the **participating CHILD** in **block letters**

____ ____________________________________

Surname, first name **of the parent or legal guardian** in **BLOCK**

Location, Date

____

Signature
